# Assay2Mol: Large Language Model-based Drug Design Using BioAssay Context

**Published:** 2025-07-16

**Authors:** Yifan Deng, Spencer S. Ericksen, Anthony Gitter

**Affiliations:** 1 Department of Computer Sciences, University of Wisconsin-Madison; 2 Morgridge Institute for Research; 3 Drug Development Core, Small Molecule Screening Facility, University of Wisconsin Carbone Cancer Center, University of Wisconsin-Madison; 4 Department of Biostatistics and Medical Informatics, University of Wisconsin-Madison

## Abstract

Scientific databases aggregate vast amounts of quantitative data alongside descriptive text. In biochemistry, molecule screening assays evaluate the functional responses of candidate molecules against disease targets. Unstructured text that describes the biological mechanisms through which these targets operate, experimental screening protocols, and other attributes of assays offer rich information for new drug discovery campaigns but has been untapped because of that unstructured format. We present Assay2Mol, a large language model-based workflow that can capitalize on the vast existing biochemical screening assays for early-stage drug discovery. Assay2Mol retrieves existing assay records involving targets similar to the new target and generates candidate molecules using in-context learning with the retrieved assay screening data. Assay2Mol outperforms recent machine learning approaches that generate candidate ligand molecules for target protein structures, while also promoting more synthesizable molecule generation.

## 1 Introduction

Early-stage drug development and target validation typically involve a search through chemical space for drug-like molecules that perturb a target of interest, usually a protein activity connected to a disease condition. For a new target, the search starts with assay development and scaling for high-throughput testing in order to experimentally screen a pre-defined chemical library for active molecules. From data obtained on screens of a target of interest (or related targets), computational models can learn structure-activity relationships that inform selection of new molecules for testing.

Public screening data repositories like PubChem (Kim et al., 2024) and ChEMBL (Mendez et al., 2018) possess great value in this regard. PubChem now contains 1.77 million assay records (BioAssay records) comprising ∼300 million bioactivity outcomes across ∼250,000 protein targets and 1,000s of cell lines^1^. BioAssay records are richly annotated with chemicals tested, target genes, pathways, proteins, cell lines, publications, patents, related BioAssay records, tabular molecule testing results, text descriptions of assay format, protocols, and relevance to disease states.

Sorting through these repositories for the data pertinent to an arbitrary target is a daunting task. Scientists need workflows that can rapidly identify relevant BioAssay records based on their associated text, extract key textual and tabular chemical testing data comprising molecule structures paired with experimental activity outcomes, and apply this information to models capable of learning structure-activity relationships to recommend new molecules for testing.

Given the extensive descriptive text components in BioAssay records, leveraging natural language processing capabilities becomes crucial for efficient retrieval and interpretation. Large language models (LLMs), with their advanced ability to process and interpret unstructured text, are well-suited for assessing BioAssay relevance by extracting key experimental details and identifying meaningful activity patterns. LLMs have demonstrated great ability in different kinds of tasks including translation, multi-round conversation, and so on. LLMs are also adept in biology and chemistry related tasks, for example, molecule property prediction, and text-guided molecule generation. LLMs support in-context learning, which extends to scientific domains. GPT-3 (Brown et al., 2020) highlights the ability of LLMs to adapt to new tasks with minimal examples. In text-guided molecule design, GPT-4 outperforms other fine-tuned models with few-shot in-context learning. (Zhao et al., 2024; Guo et al., 2023) This raises the question of whether LLMs can support different strategies for molecule design. Beyond generating molecules that satisfy specified property constraints, can LLMs navigate unstructured text within public BioAssay records and then use them as context to design molecules with functional properties, such as protein inhibition?

**Figure 1:**
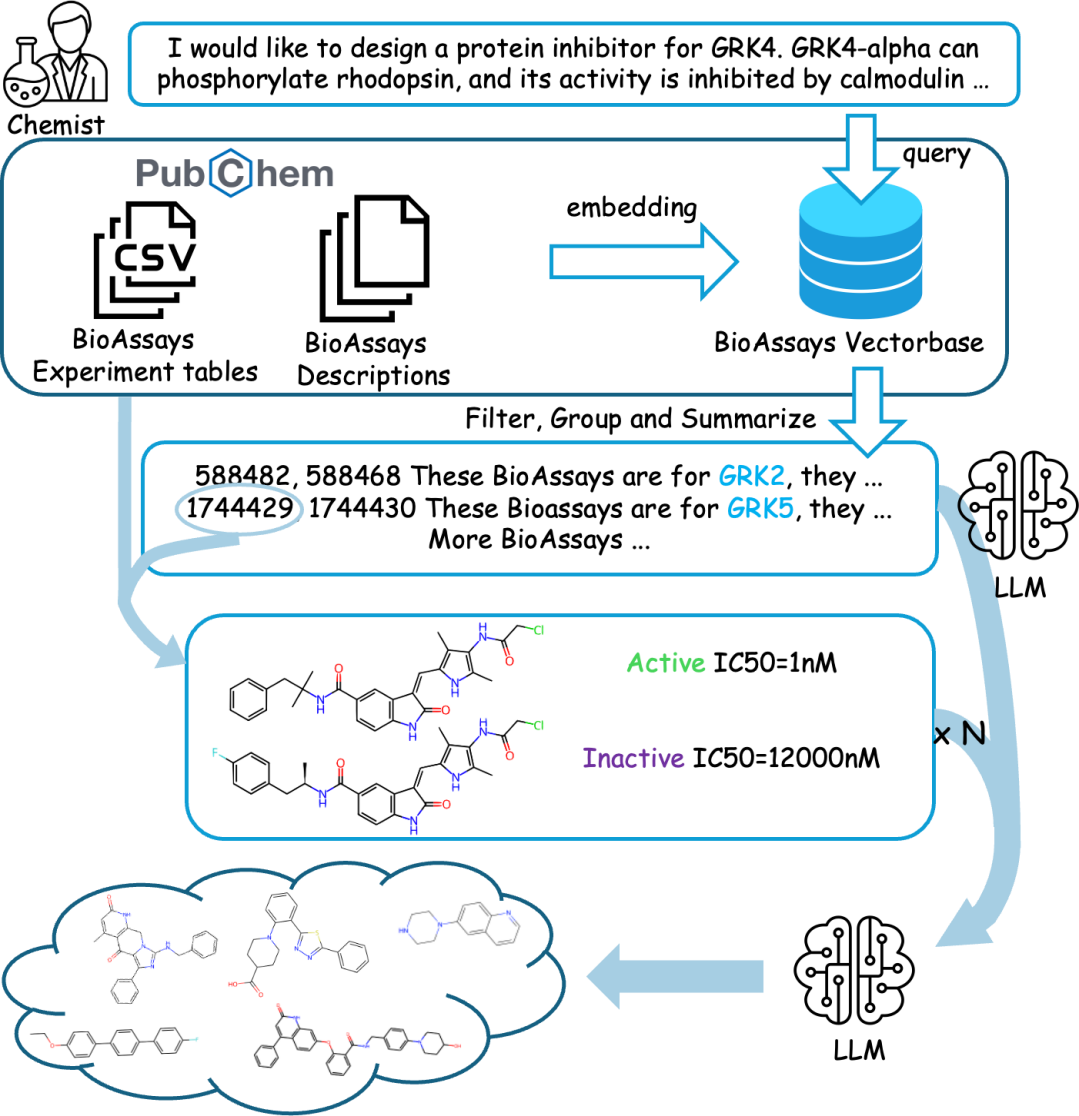
The Assay2Mol workflow. A chemist provides a target description, which is used to retrieve BioAssays from the pre-embedded vector database. After filtering for relevance, the BioAssays are summarized by an LLM. The BioAssay ID is then used to retrieve experimental tables. The final molecule generation prompt is formed by combining the description, summarization, and selected test molecules with associated test outcomes, enabling the LLM to generate relevant active molecules. The icons come from Flaticon.com and svgrepo.com

We propose Assay2Mol to maximize the use of BioAssay records with LLMs. Given input such as protein target descriptions, phenotypic data, or other textual information, Assay2Mol retrieves relevant BioAssays and leverages in-context learning to generate new molecules with the desired biological activity (Figure 1). Our contributions can be summarized as follows:

- We introduce Assay2Mol, an LLM-based drug design workflow that retrieves relevant PubChem BioAssay data for a given query and then learns to generate new candidate molecules from this assay context.
- Unlike structure-based drug discovery (SBDD) methods, Assay2Mol does not require protein structures or even sequences. It can even generate active molecules for cell-based and phenotypic assays and endpoints (e.g., tumor shrinkage, cardiotoxicity, QT interval prolongation).
- Because Assay2Mol relies on LLMs that are pretrained on chemical data for its molecule generation, the output molecules can be more like “retrieval” rather than *de novo* “generation”. This makes the generated molecules chemically plausible and synthetically accessible.

## 2 Related work

### 2.1 Large Language Models in biochemistry

LLMs are an alternative to biomolecular sequence or structure-based models for learning structure-activity or structure-property relationships. Multimodal molecule and text generation (Edwards et al., 2021; Pei et al., 2024; Deng et al., 2024; Zhao et al., 2024; Fang et al., 2023), in-context learning for chemistry (Fifty et al., 2023; Jablonka et al., 2024; Nguyen and Grover, 2024; Moayedpour et al., 2024; Schimunek et al., 2025), and LLM agents (M. Bran et al., 2024; McNaughton et al., 2024; Swanson et al., 2024; Gao et al., 2025; Liu et al., 2025; Wei et al., 2025) are at the frontier of this interface of LLMs and biomolecules. Recent reviews (Zhang et al., 2024; Mirza et al., 2024; Ramos et al., 2025; Wang et al., 2025) provide broader coverage of this expansive area.

### 2.2 BioAssay data mining

BioAssay databases like PubChem and ChEMBL are valuable resources for data mining and have been used to train machine learning models. Sharma et al. (2024) developed a data mining pipeline that compiled and processed 8,415 OXPHOS-related BioAssays from PubChem, identified major OXPHOS inhibitory chemotypes, and trained effective OXPHOS inhibitor classifiers. MolecularGPT (Liu et al., 2024) constructed an instruction tuning dataset by collecting three-shot examples from ChEMBL. MBP (Yan et al., 2023) created a multi-task pretraining dataset with labels from BioAssays to address label inconsistencies and data scarcity. TwinBooster (Schuh et al., 2024) developed a zero-shot model that integrates molecule structures and BioAssay descriptions for molecular property prediction.

### 2.3 Structure-based ligand design

Molecule design approaches play a role in the hit-finding and lead optimization tasks of early-stage drug discovery. The goal of hit finding is to identify pharmacologically active molecules for a target of interest to serve as starting points for development. Given availability of a 3D structure model for a protein target, structure-based virtual screening methods can be applied for hit finding, such as large-scale docking of pre-enumerated molecule libraries. Alternatively, *de novo* design approaches (Bohacek et al., 1999; Spiegel and Durrant, 2020) have reemerged with the advent of deep learning-based generative methods adapted to build molecules with enhanced affinity for a target structure of interest. A variety of generative approaches have been explored, including Conditional Variational Autoencoder (cVAE) (Ragoza et al., 2022), flow-based models (Shi et al., 2020; Jiang et al., 2024; Qu et al., 2024; Cremer et al., 2025), diffusion models (Guan et al., 2023; Schneuing et al., 2024; Guan et al., 2024), and Generative Pretrained Transformers (GPTs) (Wu et al., 2024).

## 3 Assay2Mol framework

### 3.1 Motivating example

**Figure 2:**
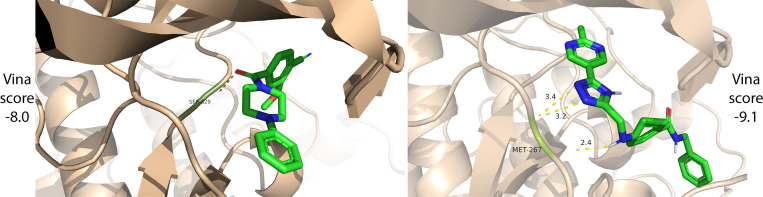
Docked binding poses of generated molecules without (left) and with (right) BioAssay context. With BioAssay context, ChatGPT 4o generates a molecule with three hydrogen bonds to the GRK4 pocket residue MET-267, improving the docking score.

Before designing a general algorithm, we begin with a proof of concept to assess whether an LLM generates relevant molecules and how BioAssay context affects generation. Using the UniProt (The UniProt Consortium, 2023) protein description of GRK4 (UniProt P32298; PDB 4YHJ) as input, we prompt ChatGPT 4o to generate five molecules. Next, we use the same protein description to search for related BioAssays (see Section 3.3) and retrieve four BioAssays related to GRK4: 775998^2^ (human GRK2), 1315729 (human GRK2), 775996 (human GRK5), 1315749 (bovine GRK2). We generate another five molecules, this time providing the retrieved BioAssay data, including the experimental tables, as additional context to ChatGPT 4o. The average AutoDock Vina (Eberhardt et al., 2021) score for the five molecules without BioAssay context is −7.44 (expressed in units of kcal/-mol). The average Vina Dock score for the four molecules with BioAssay context is −8.48 (one was invalid). Lower scores reflect better structural complementarity between the generated molecule and the protein target. Docked poses for the top scoring molecule from each group are shown in Figure 2. This small pilot study supports the proposition that the BioAssay context can improve molecule generation and motivates a full exploration of that problem setting.

### 3.2 Problem definition

Given a text description p for a target protein or phenotype, we want to retrieve the most relevant BioAssays b. Then, based on the experimental results associated with the BioAssays b, we want to generate molecules that produce the desired target response or activity. Below we use “query protein”, though it could be a target protein or phenotype.

### 3.3 BioAssay retrieval

The BioAssay retrieval stage is similar to Retrieval Augmented Generation (RAG) (Lewis et al., 2021). For a query description, we extract keywords with an LLM and obtain a protein description embedding $\text{p}\in {\mathbb{R}}^{d}$. We use the OpenAI text embedding tool (Neelakantan et al., 2022) and obtain an embedding for BioAssay record i in json format, recorded as ${\text{b}}_{i}\in {\mathbb{R}}^{d}$ (Douze et al., 2025). We use cosine similarity to calculate the similarity between the query protein description and the set of available PubChem BioAssays and then select the top-k related BioAssays:

(1) $$I_{k}=\text{arg top-}k\left\{\frac{\text{p}\cdot {\text{b}}_{i}}{\|\text{p}\|\left\|{\text{b}}_{i}\right\|}:i=1,2,\ldots ,N\right\}\;\left\{{\text{b}}_{i}:i\in I_{k}\right\}.$$

In contrast to RAG, we do not use the retrieved BioAssays as context directly. Instead, we download data tables of these BioAssays based on their Assay ID (AID) and perform further filtering:

- To ensure fair comparisons, BioAssays directly involving the query protein, identified by matching UniProt IDs, are excluded.
- Many BioAssays are derived from literature and involve only a single or few molecules. We prioritize BioAssays with larger data tables using a filter threshold *min*_*mol*_*num*, which removes BioAssays with fewer molecules tested.
- More shots will usually lead to better performance with in-context learning. However, the context length of an LLM is limited. Thus, we set *max*_*assay*_*num* to limit the number of BioAssays retrieved.
- Sometimes the query protein has no relevant BioAssays. In such cases, the retrieved BioAssays with the highest cosine similarity would be uninformative. To further assess relevance of the retrieved BioAssays, we also use an LLM to determine whether the retrieved BioAssays are relevant to the query protein.

### 3.4 Counterscreen BioAssay

BioAssay records are sometimes grouped by project. Projects contain independent records for primary screens (often high-throughput screens) and confirmatory screens (secondary re-testing of hits from the primary screen). Of particular interest are counterscreens, which are designed to detect false positives such as pan-assay interference compounds (PAINS) (Baell and Nissink, 2018) or assess hit specificity by testing for activity on an off-target or “anti-target,” an undesirable, sometimes related target. Active molecules in counterscreens are undesirable and should be avoided or used as negative training instances. Therefore, we use the LLM to summarize the retrieved BioAssays and identify whether the BioAssay record represents a counterscreen assay (Appendix A.9.1).

### 3.5 Layered contextual analysis

The LLM sequentially processes the set of relevant BioAssay records to build an input prompt for molecule generation. The workflow processes each BioAssay in three steps:

1. **Summarization of BioAssay findings:** The LLM generates a concise summary that captures the purpose, methodology, and key results of each BioAssay. Additionally, the LLM states the apparent relationship between the BioAssay and the query protein, considering how the BioAssay’s findings may inform the design of new molecules. If the BioAssay is a counterscreen, its active molecules should be avoided.
2. **Presentation of tabular experimental data:** For each BioAssay, the experimental data are presented in a table describing the SMILES notation of each molecule and its activity result (Active, Unspecified, or Inactive). Most experimental data tables also include measured pharmacodynamic parameters expressed using standard types (e.g., $IC_{50}$, $K_{i}$, $K_{d}$, percent inhibition), relations (e.g., <, =, >), values, and units (e.g., μM, %).
3. **Molecule selection:** If active molecules are identified in the data table:
  - If the number of actives exceeds $N_{mol}$, randomly sample $N_{mol}$. Otherwise list all active molecules.
  - To maintain class balance, randomly sample $N_{mol}$ molecules from the combined unspecified and inactive categories.
  If there are no active molecules, we include all molecules unless they exceed $2\cdot N_{\text{mol}}$, in which case we randomly sample $2\cdot N_{\text{mol}}$. Accordingly, we increase *min*_*mol*_*num* to $2\cdot N_{\text{mol}}$ to account for the lack of actives.

An example of the summarized BioAssays is listed in Appendix A.8.

### 3.6 Molecule generation

Given the context of the query protein description and BioAssay summaries and data tables, Assay2Mol uses the LLM to generate molecules in batches of 10. Details are in the prompt in Appendix A.9.2. The full Assay2Mol workflow is shown in Figure 1.

## 4 Experiments

We evaluate Assay2Mol in two settings. First, we compare Assay2Mol with SBDD methods for generating candidate protein-binding molecules. Second, we examine Assays2Mol’s ability to manage multiple objectives by generating molecules that bind a query protein and avoid cardiotoxicity.

### 4.1 Generating binders for target proteins Dataset.

CrossDocked2020 (CrossDocked for short) is a common dataset for SBDD (Francoeur et al., 2020) that allows us to assess how BioAssay context compares to protein structure context for generating candidate protein binders. Previous methods refined the original 22.5 million docked protein binding complexes by isolating those with poses < 1Å RMSD from native (crystallographic poses) and sequence identities < 30% from the original dataset. They used 100,000 complexes for training and 100 novel complexes as references for testing. Assay2Mol does not require additional training. We select 100 complexes from the training set to develop our pipeline and then evaluate on the test set. As input prompts for protein targets, we use the descriptions returned from PubChem from queries of the PDB ID of each protein. For proteins whose PDB ID cannot be found in PubChem, we use the UniProt mapping tool to convert the PDB ID into the UniProt ID, which is then used to query the PubChem protein webpage. When this approach fails, we manually collect information about the protein from the literature listed on its PDB homepage (Burley et al., 2023).

#### Methods.

Before evaluating Assay2Mol on CrossDocked, we selected its hyperparameters using nine proteins from the CrossDocked training set. We set *max*_*assay*_*num* to 10, $N_{mol}$ to 8, and *max*_*mol*_*size* to 45. We filter out molecules greater than *max*_*mol*_*size* from the input context in the BioAssay data table, which helps control the size of the generated molecules. We test Assay2Mol with three LLMs: Gemma-3–27B (Gemma Team et al., 2025), DeepSeekV3 (DeepSeek-AI et al., 2024), and GPT 4o (OpenAI et al., 2024). Details are in Appendix A.2.

We compare Assay2Mol against the following SBDD methods: CVAE (Ragoza et al., 2022), AR (Luo et al., 2022), Pocket2Mol (Peng et al., 2022), GraphBP (Liu et al., 2022), TamGen (Wu et al., 2024) and TargetDiff (Guan et al., 2023). Among these, TamGen generates SMILES, whereas the other methods generate 3D molecule conformations. We obtain the previously generated TamGen results from its repository. Docking scripts and other methods’ results come from the TargetDiff repository. When rerunning the docking and evaluation scripts, some results may differ from those reported in previous papers due to differences in computing environments. We prioritized comparing to these SBDD methods that had readily available generated molecules or scripts.

The Gemma-3–27B, GPT 4o, and DeepSeekV3 methods are an Assay2Mol ablation that assesses how much of the molecule generation capability comes from the BioAssay context. These methods generate molecules with an LLM using the protein description alone, as shown in Appendix A.9.4.

#### Metric.

We use **Vina Dock** to score the binding complementarity of a molecule, or the strength of interaction, with a protein target. **High Affinity** indicates the percentage of generated molecules that outperform the reference molecules in **Vina Dock**. Qualitative Estimate of Drug-Likeness (**QED**) and Synthetic Accessibility (**SA**) are computed with RDKit (Landrum, 2016). The Vina score is correlated with the number of atoms in the molecule (Weller and Rohs, 2024), so we also track the **Molecule Size**. We also discuss the molecule **validity** rate and **price** for different LLMs we use in Appendix A.3.

Metrics are calculated for each protein target. First, we compute the average metrics of the generated molecules for each corresponding protein. Then, we calculate the mean and median scores across all 100 proteins. As an additional baseline that can highlight protein-specific biases in docking scores, we randomly sample 100 FDA-approved drugs.

#### Results.

All versions of Assay2Mol consistently outperform the best SBDD method, TargetDiff, in average docking scores (Table 1). Assay2Mol (Gemma-3–27B) produces better docking scores than the other Assay2Mol variants, though it generates larger molecules, making it appealing as a locally-run open weights model that can process BioAssay context. Beyond improved docking scores, Assay2Mol generates molecules with relatively high synthetic accessibility and QED scores, benefiting from LLMs’ molecule generation capability. However, the GPT 4o and DeepSeekV3 versions perform poorly in terms of molecular diversity. We find that LLMs tend to generate similar molecules within a group when context molecules are provided. In extreme cases in our preliminary testing that generated 100 molecules per batch, the model incrementally added a single carbon atom to the molecular backbone each time. The current setting of 10 molecules per batch alleviates this issue and helps balance computing costs and molecule diversity. It is surprising that the three LLMs in the Assay2Mol ablation can generate high-quality molecules in a zero-shot setting, outperforming some SBDD models. These molecules also exhibit desirable drug-likeness and synthetic accessibility characteristics (QED, SA). However, without guidance from the BioAssay context, the generated molecules tend to be smaller in size and exhibit less favorable docking scores than Assay2Mol. We also compute the similarity between generated and context molecules to demonstrate that the LLMs learn from context rather than merely making minor modifications to existing molecules. More details are shown in Appendix A.4.

A more detailed examination of the mouse protein TFPI provides context to Assay2Mol’s performance on the CrossDocked dataset (Appendix A.6). It reveals that not all BioAssays with similar embeddings to the target protein query are biologically relevant. This is why we use an LLM to estimate the relevance of the retrieved BioAssays to the query protein. For each query protein, we analyze the top 10 BioAssays after filtering. We employ GPT 4o and DeepSeek-V3 and then aggregate their results, and define x as relevant BioAssays/total BioAssays. We categorize the proteins into four groups: high relevance (x≥0.7), medium relevance (0.4<x<0.7), low relevance (0.1<x≤0.4), and no relevance (x≤0.1). The results of different groups are shown in Figure 4. As expected, Assay2Mol performs worst on the no relevance group compared with TargetDiff, and it is consistent with our small-scale evaluation of low-similarity BioAssays (Figure 9). To check the accuracy of the LLM relevance evaluation, we manually evaluate the BioAssays retrieved for 25 targets and find that GPT 4o is fairly accurate in its relevance assessment but DeepSeek-V3 struggles (Table 4). DeepSeek-V3 incorrectly assesses the relevance for all ten BioAssays for six different targets.

Since docking score distributions differ across proteins, directly averaging scores as in Table 1 may fail to capture meaningful improvements over baseline methods. To account for protein-specific effects, we dock 100 FDA-approved drugs to the 100 CrossDocked test proteins to establish a baseline score distribution. We then compute the improvement in docking score for each generated molecule with respect to these protein-specific background distributions. The improvement of Assay2Mol decreases as BioAssay relevance decreases, while TargetDiff demonstrates relatively uniform performance across all groups, consistent with its reliance solely on protein structure (Table 2). GPT 4o outperforms Assay2Mol (GPT 4o) in the low relevance group, suggesting that the inclusion of irrelevant BioAssay context may mislead the LLM for molecule generation. LLMs without context perform poorly in the no relevance group, indicating that these proteins are possibly understudied and underrepresented in existing databases and their pretraining data. SBDD is a more suitable strategy in such cases. These observations reinforce the validity of the Assay2Mol concept, suggesting that the LLM benefits from assay context to guide molecule generation. Also, most of the proteins in the CrossDocked test set fall into the “High” and “Medium” groups (81%), indicating Assay2Mol’s practical utility for potential targets of interest.

### 4.2 Specificity and counterscreen with hERG

KCNH2, also known as hERG, is a voltage-gated potassium ion channel that plays a crucial role in cardiac repolarization. Blocking hERG channels with drugs can lead to prolonged QT intervals, potentially causing severe cardiac arrhythmias or sudden death. It has been one of the most frequent adverse side effects leading to the failure of drugs (Sanguinetti and Tristani-Firouzi, 2006). As a result, evaluating a molecule’s interaction with hERG is a critical step in drug development to ensure safety. In the PubChem database, there are many BioAssays using hERG as a counterscreen (Garrido et al., 2020). We ask:

1. Can Assay2Mol accurately interpret the counterscreen context to reduce the generated molecule’s affinity for hERG?
2. Can the generated molecules retain high affinity to the original target protein?

We selected proteins GRK4 (PDB: 4YHJ), HD8 (PDB: 4RN0), and CD38 (PDB: 3DZH) from the CrossDocked test set. These proteins serve as potential targets for cancer treatments or antibiotics. Since the corresponding molecules may potentially be developed as drugs taken by humans, it is crucial to evaluate their potential hERG-related interactions to assess safety. After we generate molecules for these proteins using the same methods as in the CrossDocked evaluation, we use the description of hERG^3^ to search for related BioAssays, in the same manner used to formulate the context described in Section 3. After we prepare the whole context, we append generated molecules from the previously selected proteins and ask the LLM to optimize these molecules to reduce binding affinity toward hERG. The prompt is shown in Appendix A.9.5. We use ADMETlab 3.0 (Fu et al., 2024) to predict the hERG score. To avoid circular reasoning, we verify that no molecules in the hERG generation context were present in the ADMETlab 3.0 hERG training set.

**Figure 3:**
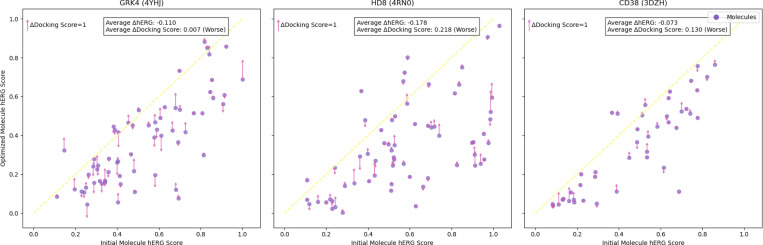
Change in predicted hERG score and docking score between initial and optimized molecules for three proteins. The up arrow indicates the docking score decreases and the down arrow indicates it increases. The length of the arrow (top-left) serves as a scale bar, representing an increase of 1 score unit (kcal/mol) from Vina Dock.

We examine the shift in Vina docking scores and predicted hERG scores for the original generated molecules versus those optimized to minimize hERG interaction (Figure 3) The average hERG score of molecules for 4YHJ decreases from 0.503 before optimization to 0.393 after optimization. For comparison, the average hERG score of 2,965 FDA-approved drugs is 0.284. This reduction in hERG score indicates lower predicted cardiotoxicity, making the generated molecules more suitable for further study, while docking scores remain largely unaffected, demonstrating that in-context learning with a counterscreen enhances specificity without compromising affinity.

## 5 Discussion and conclusions

Our initial version of Assay2Mol can successfully query PubChem with text descriptions, retrieve relevant BioAssays, and generate molecules based on the text and screening data from those relevant BioAssays. Assay2Mol shows how to use LLMs to make better use of decades worth of valuable, unstructured chemical screening data in PubChem. Our approach generates molecules with docking scores comparable to SBDD methods and advances controllable natural language-driven molecule design. There are many opportunities to expand on the core Assay2Mol framework, making it more robust and building new capabilities. For example, we observed that the initial embedding-based query and BioAssay similarity calculations are not sophisticated enough to capture the complexity of biological regulation in pathways (Appendix A.6) along with other limitations in Section 6.

## 6 Limitations

There are several opportunities to improve how LLMs are used within Assay2Mol. Having LLMs directly assess the relevance of the retrieved BioAssay text guards against many irrelevant matches but is imperfect (Table 4). We continue to explore methods to enhance LLM interpretation of the desired activity of generated molecules with respect to target function (i.e., activation, inhibition, allosteric regulation). A related limitation is that the current version of Assay2Mol cannot properly process conditional text queries, such as molecules that inhibit proteins A, B, and C but not D and E. This limitation is again related to the initial embedding-based similarity calculations, which could possibly be addressed with enhancement to the Assay2Mol relevance processing step. Our hERG example shows that Assay2Mol can be used for conditional molecule generation, for example inhibiting GRK4 but not hERG, if these steps are run sequentially. Furthermore, there is potential for improvement in both construction of text prompts and the LLMs used within the Assay2Mol framework. Our BioAssay relevance assessment evaluation showed that GPT 4o matched manual assessments much more closely than DeepSeek-V3 (Table 4), so the choice of LLM can impact the overall results. Finally, we have not yet evaluated the sensitivity of Assay2Mol to different LLM prompting strategies or optimized the prompts.

Most of the LLM-based steps in Assay2Mol are implemented using both closed and open weights LLMs with the goal of having a fully open weight version of Assay2Mol. However, currently the BioAssay embeddings are generated solely using the OpenAI text embedding API. An alternative implementation and evaluation with open weights embedding models remains for future work. Even the open weights LLMs Assay2Mol uses are not fully open source and do not have their training data available. This lack of training data and what biochemistry data are included makes it challenging to fully interpret the Assay2Mol-generated molecules and their limitations. If it is generating novel molecules as opposed to retrieving molecules from the LLM training set, general caveats about generative molecular design apply to those outputs (Walters, 2024).

The evaluations of the Assay2Mol generated molecules rely entirely on other computational assessments of molecule quality. These are not a substitute for actual wet lab assays. Vina Dock energies are not true binding affinities, and its scoring function has known biases and limitations (Xu et al., 2022). The hERG scores are computed with an existing regressor that has reasonably good but imperfect performance (Fu et al., 2024).

## Supplementary Material

Supplement 1

## Figures and Tables

**Table 1:** Experimental results on the CrossDocked dataset. The best results are shown in **bold**, and the second-best results are underlined.

Model	Vina Dock (↓)		High Affinity (↑)		QED (↑)		SA (↑)		Diversity (↑)		Size	
	Avg.	Med.	Avg.	Med.	Avg.	Med.	Avg.	Med.	Avg.	Med.	Avg.	Med.
Reference	−7.117	−6.905	-	-	0.476	0.468	0.728	0.740	-	-	22.75	21.50
FDA drugs	−7.027	−7.169	0.440	0.380	0.567	0.564	0.760	0.758	0.792		24.47	

CVAE	−6.114	−6.118	0.103	0.026	0.390	0.419	0.591	0.580	0.655	0.666	19.97	20.19
AR	−6.751	−6.707	0.459	0.340	0.505	0.499	0.635	0.634	0.698	0.703	17.78	17.54
Pocket2Mol	−7.200	−6.815	0.601	0.593	0.574	0.579	0.754	0.760	0.741	0.781	17.84	16.53
TamGen	−7.475	−7.775	0.526	0.645	0.559	0.559	0.771	0.759	0.747	0.745	23.13	23.29
TargetDiff	−7.788	−7.964	**0.683**	0.634	0.474	0.485	0.584	0.571	0.717	0.714	24.44	24.64

Gemma-3-27B	−7.050	−7.024	0.416	0.281	0.700	0.711	0.860	0.868	0.757	0.765	19.34	18.94
GPT 4o	−7.198	−7.257	0.432	0.294	**0.789**	**0.803**	**0.870**	**0.878**	0.767	0.767	19.70	19.64
DeepSeekV3	−7.230	−7.170	0.443	0.241	0.743	0.756	0.855	0.867	**0.771**	**0.772**	18.96	19.00

Assay2Mol (Gemma-3-27B)	**−8.064**	**−8.280**	0.610	**0.732**	0.585	0.606	0.821	0.834	0.742	0.611	26.59	26.65
Assay2Mol (GPT 4o)	−7.796	−7.881	0.548	0.576	0.600	0.630	0.790	0.801	0.542	0.547	25.90	25.59
Assay2Mol (DeepSeekV3)	−7.861	−7.936	0.557	0.634	0.616	0.647	0.813	0.820	0.593	0.608	24.46	24.26

**Table 2:** Average improvement over randomly sampled FDA drugs grouped by LLM-estimated relevance of the retrieved BioAssays. The value represents the increase in the docking score, measured in kcal/mol.

Model	High (39%)		Medium (42%)		Low (7%)		No (12%)		Overall	
	Avg.	Med.	Avg.	Med.	Avg.	Med.	Avg.	Med.	Avg.	Med.
TargetDiff	0.838	0.802	0.701	0.777	0.669	0.696	0.771	1.052	0.761	0.796

Gemma-3-27B	0.196	0.170	−0.050	−0.145	0.197	0.293	−0.390	−0.535	0.023	0.034
GPT 4o	0.331	0.228	0.116	0.118	0.396	0.302	−0.289	−0.122	0.171	0.130
DeepSeekV3	0.379	0.311	0.079	0.070	0.429	0.300	−0.067	−0.098	0.203	0.159

Assay2Mol (Gemma-3–27B)	1.277	1.124	1.037	1.121	0.535	0.770	0.554	0.606	1.037	1.069
Assay2Mol (GPT 4o)	1.061	1.046	0.732	0.741	0.223	0.517	0.269	0.151	0.769	0.777
Assay2Mol (DeepSeekV3)	1.042	0.634	0.842	0.921	0.599	0.579	0.267	0.273	0.834	0.849

## Footnotes

1
https://pubchem.ncbi.nlm.nih.gov/docs/statistics

2
https://pubchem.ncbi.nlm.nih.gov/bioassay/775998

3
https://pubchem.ncbi.nlm.nih.gov/gene/3757
