# Supplementary material for: Assay2Mol: Large Language Model-based Drug Design Using BioAssay Context
Source: ArXiv. 2025 Jul 16:arXiv:2507.12574v1. Preprint. [Version 1] (PMC12288650)
Supplement: Supplementary file 1 [file NIHPP2507.12574v1-supplement-1.pdf]

A Appendix

A.1 Potential risks

Like many other molecule generation tools, Assay2Mol could be considered a dual-use technology. Its primary intended application is in early stage drug discovery as a strategy to sift through unstructured experimental BioAssay descriptions in PubChem. However, BioAssays in PubChem used for counterscreening molecules measure toxicity, so it could be possible to use Assay2Mol to generate toxic molecules. Existing generative models can also be trained or guided with this public toxicity data, so this is a risk of a broad class of models not Assay2Mol specifically.

Assay2Mol generates molecules tailored to activity against the target protein or phenotype. Because the prompts are constructed in a structured manner using BioAssay context, it is less likely to be able to generate some classes of harmful molecules like explosives compared to generative models that take free text descriptions of molecule properties as input. LLMs perform the actual molecule generation, and we have not formally studied how the BioAssay context expands or reduces risk relative to the baseline LLMs. We anticipate that Assay2Mol's structured prompts restrict the types of molecules the LLMs can generate, and some of the generated molecules are actually retrieved existing molecules.

A.2 Hyperparameter and model selection

For the Assay2Mol hyperparameter selection, we choose nine proteins from the randomly sampled 100 proteins from the training set: 1RQP, 3ANT, 4A9S, 4I29, 4XE6, 4Y2R, 5ACC, 5AI4, 5PNX. We manually inspect their retrieved BioAssays and make sure they are relevant. We also include the reference molecule and label it as "CrossDocked". Assay2Mol invloves three hyperparameters: max_assay_num, N_{mol}, and max_mol_size, making it time-consuming to perform grid-search. To simplify the process, we fix two hyperparameters when optimizing the remaining one.

First, we set N_{mol} to 5, impose no limit on max_mol_size, and vary max_assay_num across [1, 5, 10, 15, 20, 30]. According to Figure 5, we observe that $max\_assay\_num = 10$ yields strong performance. More than 10 BioAssays leads to longer context and increases computational cost without significant gains.

After max_assay_num is fixed to 10, we vary N_{mol} across [1, 3, 5, 8, 12] and find $N_{mol} = 8$ yields the best results, as shown in Figure 6. Finally, we fix max_assay_num to 10 and N_{mol} to 8 and examine max_mol_size's influence on the generated molecules. We do not explicitly prompt the LLM to generate molecules of a specific size. The generated molecules' size and the docking score both correlate with max_mol_size, as shown in Figure 7. Therefore, we set max_mol_size to different values as a way to approximately control the generated molecules' sizes. Additional figures used to guide the hyperparameter tuning are available in our GitHub repository.

For LLM selection, we first select GPT 4o as the base model, a closed weights model. Then, we select DeepSeekV3, an open weights model, so that Assay2Mol does not rely solely on proprietary LLMs. Additionally, we incorporate Gemma-3-27B, a high-performing open weights model that can be locally deployed on a single GPU. For the motivating example, we use the ChatGPT 4o web version. For GPT 4o, we use the version "chatgpt-4o-latest" (as of May 2025) for Assay2Mol experiments and version "gpt-4o-2024-11-20" for LLM BioAssay relevance assessment (Table 4), which was completed earlier. For DeepSeekV3, we use the version "DeepSeek-V3-0325" for Assay2Mol and "DeepSeek-V3-1226" for relevance assessment (Table 4). For Gemma-3-27B, we run the 4-bit bitsandbytes quantized version provided by unsloth[4] on a single RTX 5090 GPU.

A.3 LLM molecule validity and cost

Though DeepSeekV3 outperforms GPT 4o in Table 1, DeepSeekV3 generates far more invalid molecules than GPT 4o. We define validity as the percentage of unique generated SMILES that are parsable by RDKit. We list the validity and API price of different LLMs in Table 3.

A.4 Similarity analysis

Given the molecules provided in the BioAssay context, one natural question arises: Does the LLM simply copy and slightly modify the active molecules, rather than generating novel ones? To address this question, we dock the context molecules against five proteins (4AAW, 4YHJ, 14GS, 2V3R, 4RN0) and analyze the similarity between the generated molecules and the context molecules. We select context molecules

[4] https://huggingface.co/unsloth/gemma-3-27b-it-unsloth-bnb-4bit

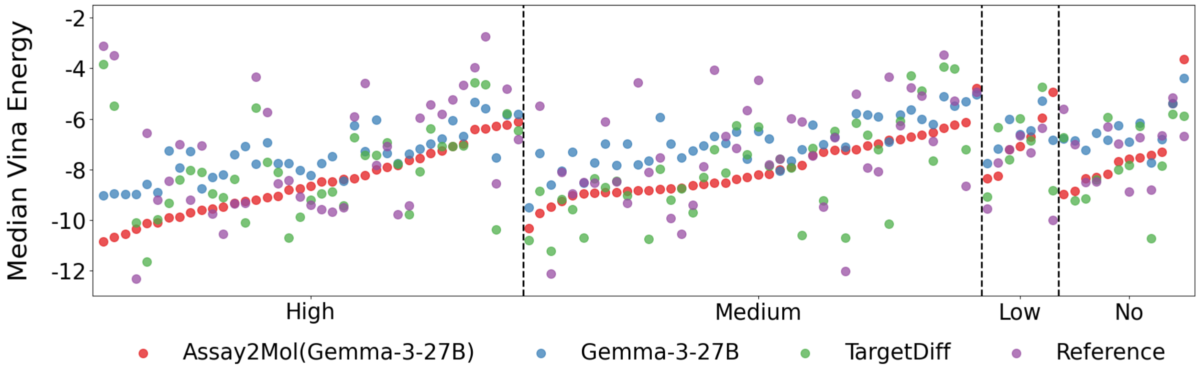

Figure 4: Distribution of docking scores of different relevance level groups.

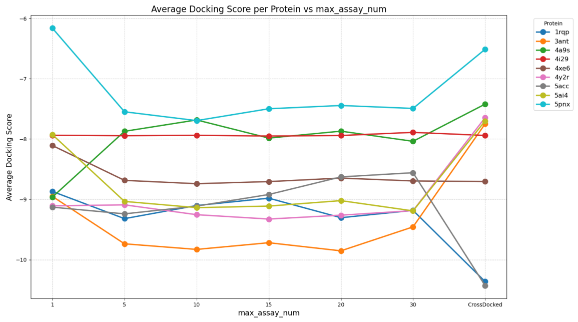

Figure 5: Average docking score under different max_assay_num

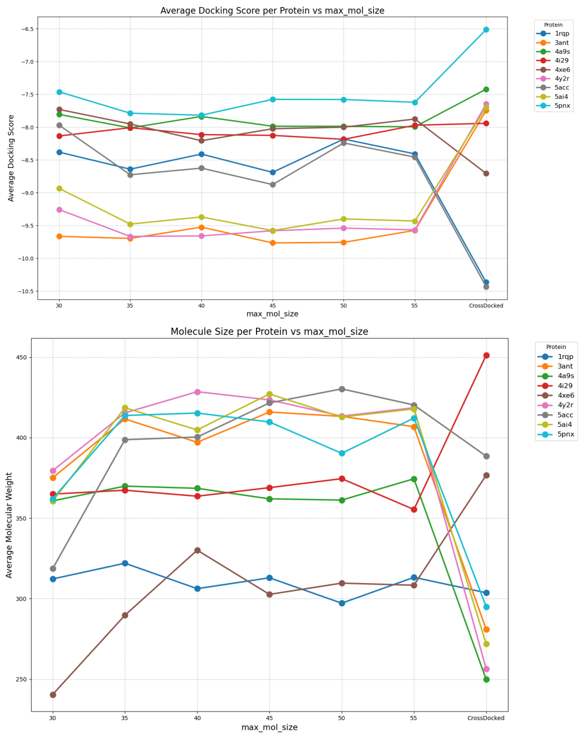

Figure 7: Average docking score and molecule weight under different max_mol_size

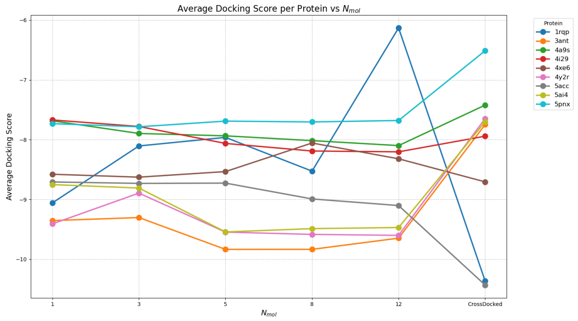

Figure 6: Average docking score under different N_{mol}

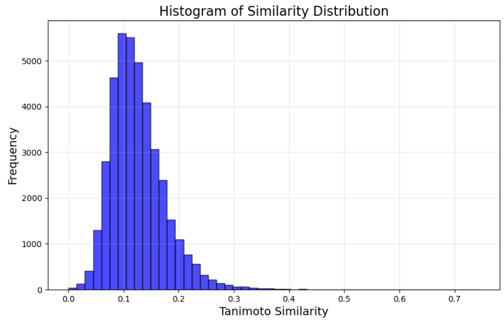

Figure 8: Similarity distribution between generated molecules and high docking score context molecules.

LLM	Validity	Input Price	Output Price
Gemma-3-27B	84.32	N/A	N/A
DeepSeekV3	75.84	\$0.07/\$0.27	\$1.1
GPT 4o	94.33	\$2.5/\$5	\$20

Table 3: Validity of generated molecules and API price of different LLMs. The price is for 1M input/output tokens. The numbers in input price represent the cost of generation with a cache hit and cache miss, respectively. The price for Gemma-3-27B is not applicable because it is run on a local machine.

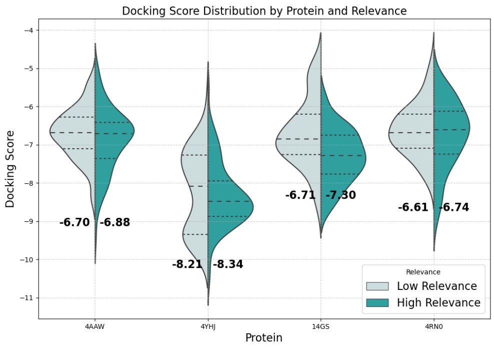

Figure 9: Distribution of docking scores across four proteins using high- and low-relevance BioAssays as context. The average docking score is shown on the top of each plot.

with docking scores more favorable than the reference molecules and consider them to be high-scoring context molecules. Then, we compute the Tanimoto similarity between all pairs of the high-scoring context molecules and the generated molecules using Morgan fingerprints as features (Figure 8). Most generated molecules are dissimilar to the high-scoring context molecules, suggesting that the LLM is learning from the context rather than simply making slight modifications to the context molecules.

A.5 BioAssay context relevance experiment

Table 2 already shows that Assay2Mol performs worse on proteins that do not have relevant BioAssays. We further investigate the impact of context relevance by substituting relevant BioAssays with irrelevant ones. Specifically, we select four proteins for which relevant BioAssays can be retrieved: PDB IDs 4AAW, 4YHJ, 14GS, and 4RN0. During the retrieval stage, we replace the BioAssays with those having relevance scores (measured in cosine similarity) ranging from 0.4 to 0.5, scores much lower than the relevant BioAssays, while keeping the rest of the Assay2Mol pipeline unchanged. The result is shown in Figure 9. Although high-relevance BioAssays consistently outperform low-relevance BioAssays on average, the difference is not particularly pronounced. We suspect this is because GPT 4o also demonstrates the capability to generate molecules solely based on protein descriptions.

To investigate further, we analyzed the context molecules from the irrelevant BioAssays selected by GPT 4o as templates and examined their docking scores toward the query protein. Across the targets 4AAW, 4RN0, 14GS, and 4YHJ the average value of the docking scores are -6.61, -6.04, -5.91, and -6.69, respectively. Even when the BioAssays were irrelevant to the query protein, these context molecules can exhibit moderately high docking scores, making it less surprising that molecules generated from that context yield comparable docking scores.

However, we noticed that the lower tails of the violin plots representing high-relevance molecules tend to extend longer than those of the low-relevance group (Figure 9), indicating that the best docking scores are more frequently found among high-relevance molecules. When using docking for hit identification, researchers focus on the top-scoring molecules as opposed to the entire distribution because these are most likely to be enriched for actual active molecules. Thus, we extended the experiment to 18 proteins and only focused on the molecules with the 10 best docking scores per model. We found that in 11 out of 18 proteins, the high-relevance group performed better than both the low-relevance group and the without context group, which is the GPT 4o baseline (Figure 10). In five proteins (4YHJ, 3DAF, 3DZH, 1COY, 2PQW), the high-relevance group performed similarly to other groups. In two proteins (2JJG, 3GS6), the high-relevance group performed worse than other groups.

These results suggest that in some cases, molecules derived from low-relevance BioAssays can still achieve favorable docking scores either by chance or due to biases in the docking software. Nonetheless, molecules from high-relevance BioAssays generally demonstrate superior performance when focusing on the tail of the docking score distribution.

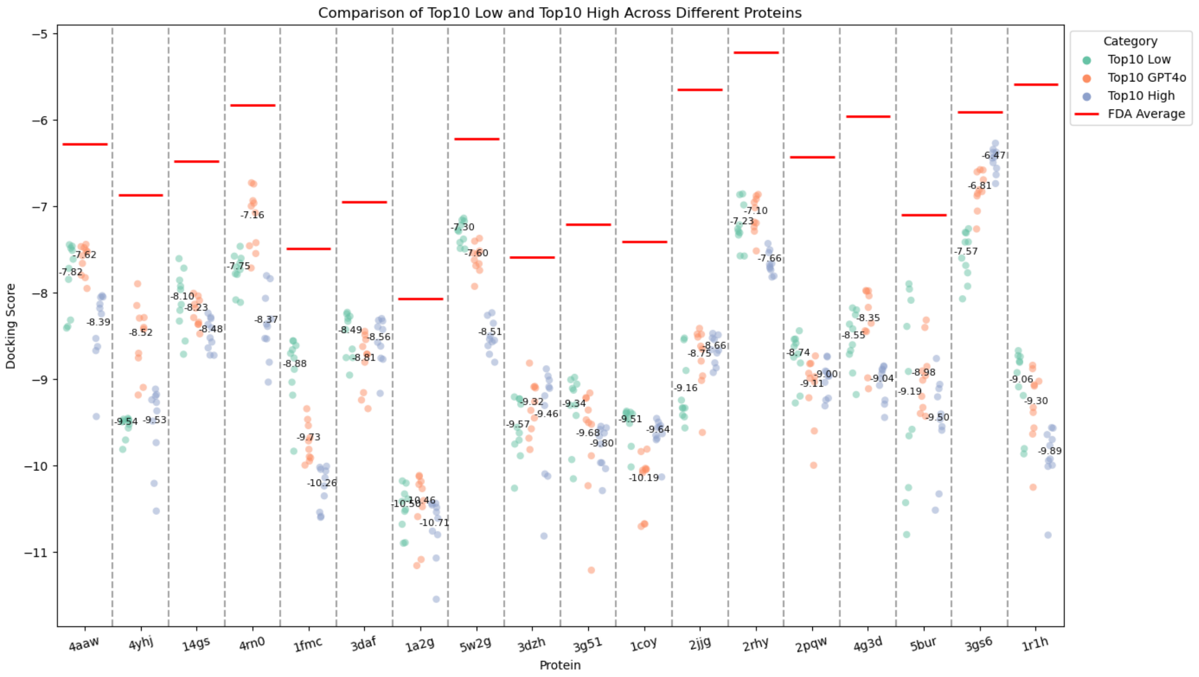

Figure 10: Distribution of the top 10 docking scores from molecules with high- and low-relevance BioAssays as context for different proteins.

A.6 Antagonistic protein pair

During the exploration of BioAssay retrieval, we found a scenario that is beyond the current capabilities of Assay2Mol. We tested Assay2Mol with recent, real drug targets by selecting the most recently FDA-approved drug of 2024[5], concizumab-mtci. This drug is an inhibitor of the human Tissue Factor Pathway Inhibitor (TFPI)[6] protein. Using the protein description of TFPI from PubChem, we performed the BioAssay retrieval task. The top-matching BioAssay[7] related to Tissue Factor (TF) and Coagulation Factor VII (FVII). Part of the retrieval result is shown in Table 5. TFPI, TF, and FVII are key regulators of the extrinsic coagulation pathway, which is responsible for initiating blood clotting (McVey, 1999). TF initiates coagulation, FVII promotes the process, and TFPI inhibits it, maintaining the dynamic balance of the coagulation system. TF inhibitors are designed to treat hypercoagulability and thrombosis. TFPI inhibitors are used for treating hemophilia A and B. Since TF and TFPI have antagonistic functions, an incorrect selection of BioAssays leads to misaligned molecule design. When designing an inhibitor for TFPI, retrieving BioAssays targeting TF directs the molecule design in the opposite direction. Among the 300 retrieved BioAssays, only four directly relate to TFPI, and their target protein is mouse TFPI. The majority are associated with TF, the functional opposite of TFPI.

Although reasoning LLMs (for example, GPT o1 and DeepSeek R1) recognize the antagonistic relationship between the retrieved BioAssays and the query protein—allowing them to discard most mismatched results—the process is not completely accurate. Some of the 296 TF BioAssays are still incorrectly labeled as relevant to TFPI, representing false positives. The LLMs still learn from inhibitors of the opposing protein, leading to failure in molecule design. While the CrossDocked dataset contains no such cases, developing a more robust retrieval pipeline remains a priority for future work.

A.7 Software versions

We use the hERG Blocker classifier from the ADMETlab 3.0 web server (Fu et al., 2024) to predict the hERG score, which is in the range [0,1].
We use AutoDock Vina (Eberhardt et al., 2021) v1.5.7 as the docking software.

[5] https://www.fda.gov/drugs/novel-drug-approvals-fda/novel-drug-approvals-2024

[6] https://pubchem.ncbi.nlm.nih.gov/protein/P10646

[7] https://pubchem.ncbi.nlm.nih.gov/bioassay/1381622

Relevance	Target	GPT 4o errors	GPT 4o accuracy (%)	DeepSeek-V3 errors	DeepSeek-V3 accuracy (%)	GPT 4o accuracy − DeepSeek-V3 accuracy (%)
high	5W2G	1	90	1	90	0
high	3G51	4	60	10	0	60
high	1COY	3	70	9	10	60
high	2JJG	5	50	9	10	40
high	2RHY	1	83	3	50	33
high	2PQW	0	100	2	67	33
high	4G3D	5	50	5	50	0
medium	4AAW	4	60	6	40	20
medium	4YHJ	0	100	1	90	10
medium	14GS	1	90	9	10	80
medium	4RN0	1	90	4	60	30
medium	1FMC	0	100	10	0	100
medium	3DAF	0	100	10	0	100
medium	1A2G	0	100	10	0	100
medium	3DZH	0	100	8	20	80
medium	5BUR	0	100	10	0	100
low	1R1H	0	100	7	30	70
low	5B08	0	100	3	70	30
low	5I0B	1	90	4	60	30
low	3KC1	0	100	4	60	40
low	1D7J	1	90	4	60	30
no	2Z3H	0	100	0	100	0
no	2V3R	0	100	0	100	0
no	3B6H	8	20	10	0	20
no	4P6P	0	100	0	100	0
**total**	25	35	86	139	43	43

Table 4: Manual review of LLM BioAssay relevance assessment. All targets have 10 BioAssays retrieved except for 2RHY and 2PQW, which have 6 each. The reason is there are only 6 BioAssays remained after filtering.

BioAssay	Score	Target	Relationship
1381622	0.8284	Tissue factor, Coagulation factor VII	Opposite
51307	0.8245	Tissue factor, Coagulation factor VII	Opposite
397857	0.8233	Tissue factor, Coagulation factor VII	Opposite
385437	0.8202	Tissue factor	Opposite
385438	0.8194	Tissue factor	Opposite
385435	0.8190	Tissue factor	Opposite
385436	0.8188	Tissue factor	Opposite
1871821	0.8183	Coagulation factor VII	Opposite
360094	0.8182	Tissue factor	Opposite
385434	0.8180	Tissue factor	Opposite
⋮			
1620662	0.7941	Tissue factor pathway inhibitor	Correct
1620663	0.7903	Tissue factor pathway inhibitor	Correct
⋮			
1476906	0.7888	Coagulation factor XII	Opposite
1775843	0.7888	Carboxypeptidase B2	Opposite
304051	0.7888	Carboxypeptidase B2	Opposite
52023	0.7888	Coagulation factor X	Opposite
1426475	0.7888	Coagulation factor X	Opposite
1775842	0.7888	Carboxypeptidase B2	Opposite
212091	0.7887	Tissue factor	Opposite
362212	0.7887	TISSUE: Plasma	Opposite
1382994	0.7886	Coagulation factor X	Opposite
1657762	0.7886	TISSUE: Plasma	Opposite

Table 5: BioAssay retrieval result using the TFPI description as input. The Score is measured with cosine similarity. Only four out of 300 BioAssays are related to TFPI while others have the opposite function in the pathway.

A.8 Assay summarization example

Summarized BioAssay Example

This BioAssay measures the inhibition of GRK5-mediated phosphorylation of rhodopsin in bovine rod outer segment membranes under white light conditions. It evaluates the efficacy of cyclic peptide inhibitors derived from the HJ loop of GRK2, providing insights into their potency and selectivity.
This is the activity data table. Each line has the SMILES, followed by activity type (active, inactive or unspecified) and the experimental value.
`[C@H](C(=O)N1)CCCCN))...(N)N)CC(C)C`
Unspecified Inhibition <5%
`CC[C@H](C)[C@@H](C(=O)O)...NC(=O)CN`
Unspecified Inhibition <5%

This BioAssay evaluates the inhibition of recombinant human GRK5 expressed in Sf9 insect cells, measuring the decrease in phosphorylation of urea-washed bovine rod outer segments in the presence of Gbetagamma subunits and [gamma-32P]-ATP.
This is the activity data table. Each line has the SMILES, followed by activity type (active, inactive or unspecified) and the experimental value.
`CC[C@H](C)[C@@H](C(=O)N...(CCCCN)N`
Active IC50 = 2100 nM
`C1=CC=C(C=C1)/C=C\2/C3=CC=CC=C3C(=O)N2`
Inactive IC50 = 60000 nM

A.9 Prompts

A.9.1 BioAssay summarization prompt

Prompt for BioAssays Summarization

Instruction: You are an expert in **BioAssay analysis** and **data extraction**. Your task is to carefully analyze the provided BioAssay JSON data and extract structured key information, including:
1. **BioAssay Summarization** – A concise summary of what this assay measures and its scientific purpose.
2. **Assay Type** – The experimental technique used (e.g., **Enzymatic Inhibition, Fluorescence Assay, SPR, Radioligand Binding**).
3. **Summary of Observations** – Important scientific insights derived from the BioAssay, including key patterns in activity, structural features affecting activity, and notable findings.
Step-by-step extraction process:
- Parse the **"descr"** section of the JSON, identifying key information about the assay. If this BioAssay is a counterscreen assay, set "CounterScreen" to "True".
- Identify the **Assay Type** by analyzing the **"name"** field.
- Extract **scientific insights** from the description and comments to create the **Summary of Observations**.
- Generate a **concise and informative summary** of the BioAssay, keeping scientific accuracy and relevance.
Output Format Return the extracted data in the following structured format:

```
json
{
  "BioAssay_Summary": "A brief but
      complete summary of what
      this assay is measuring and
      why it is important.",
  "Assay_Type": "The experimental
      method used (e.g., Enzymatic
      Inhibition, Fluorescence, SPR
      , etc.)",
  "Summary_of_Observations": "
      Scientific insights, key
      findings, and notable trends
      from the BioAssay.",
  "CounterScreen": "True" if the
      BioAssay is identified as
      CounterScreen against Query
      Protein, else "False".
}
```

Query Protein:
{Protein Description}
BioAssay JSON
{BioAssay JSON}

A.9.2 Generation prompt

Molecule Generation Prompt

Role: AI Molecular Generator and BioAssay Analyst

Profile
- **Author**: LangGPT
- **Version**: 1.1

- **Language**: English
- **Description**: An AI model specialized in analyzing BioAssay results, understanding protein-ligand interactions, and generating high-affinity molecules based on experimental data.

Skills
- Understanding **protein-ligand interactions** from experimental BioAssay data.
- Interpreting **BioAssay results** and extracting meaningful insights.
- Learning from **high-affinity molecules** in BioAssay data to generate new molecules.
- Ensuring **high binding affinity and specificity**, while avoiding **Pan-assay interference compounds (PAINS)**.
- Generating drug-like molecules that align with known **active reference compounds**.

Rules
1. **Carefully analyze** the input description of the **protein**, **BioAssay**, and **experimental results**.
2. Identify **high-affinity molecules** (low IC50/Kd values) from the **BioAssay data** as **reference molecules**.
3. Use reference molecules to **learn key functional groups and molecular scaffolds**.
4. Focus on **specificity rather than only high docking scores**.
5. **Each generated molecule should be enclosed within [BOS] and [EOS]**.
6. **Each SMILES should be numbered from 1 to 10, with one per line.**
7. Avoid **PAINS compounds** and prioritize **drug-likeness**.
8. **Do not blindly maximize molecular size**, as larger molecules may have artificially high docking scores but poor specificity.

Workflows
Step 1: Understand the BioAssay and Its Relation to the Query Protein
- The BioAssays may or may not be related to the **Query Protein**, please identify the correct Query Protein first.
- The type of **assay method** used (e.g., enzymatic, fluorescence, cell-based). - How the **assay measures protein-ligand interaction**. - The **affinity measurements** (e.g., IC50, Kd, Ki).
- Extract **key active molecules** from BioAssay results. (e.g. IC50, Ki, Kd<100nM) - Identify molecular features that contribute to **high binding affinity**.

Step 2: Learn from Active Molecules and Think Step by Step
- Extract **key functional groups** and **molecular scaffolds** from high-affinity reference molecules.
- Avoid **PAINS compounds** and prioritize **specificity**.
- Ensure molecules remain within a **reasonable drug-like chemical space**.
- Optimize molecular properties for **binding affinity and selectivity**.

Step 3: Generate 10 High-Affinity Molecules
- Use the **active reference molecules** as a learning guide, and use the **low binding affinity molecules** as negative samples.
- Each generated molecule should be optimized for **binding affinity and specificity**.
- The output format must follow this structure:
- Each **SMILES string should be enclosed in [BOS] and [EOS]**.
- Each SMILES should be **numbered from 1 to 10**, with each on a separate line.
- Avoid **PAINS compounds** and prioritize **drug-likeness**.
- Avoid generating molecules that are too large

Step 4: Justify the Molecular Selection
- Explain how the **reference molecules** influenced the molecular design.
- Describe how the **assay results** guided molecular modifications.
- Justify why these molecules should have **high binding affinity and specificity**.

—

Output Format:
1. BioAssay Understanding & Analysis

- Step-by-step reasoning about the BioAs-

say, its setup, and its relevance to the query protein

2. Selected Reference Molecules from BioAssay
- List of highly active molecules from BioAssay used as reference.

3. Generated Molecules
`[BOS] SMILES_1 [EOS]`
⋮
`[BOS] SMILES_10 [EOS]`

4. Justification for Molecular Selection
- Explanation of how reference molecules influenced design choices, ensuring specificity and affinity while avoiding PAINS.

Query Protein
{Protein Description}

BioAssays
{Assay Content}

A.9.3 Relevance assessment prompt

Relevance Assessment Prompt

Instruction:
You are an expert in **BioAssay analysis** and **data extraction**. Your task is to carefully analyze the provided BioAssay JSON data and extract structured key information, and decide whether the protein studied in the BioAssay is related to the Query Protein, with broader consideration of similarity:
- If the BioAssay can help protein inhibitor design toward the **Query Protein**, set "Relevant": "True".
- **Use a broad definition of similarity**, including:
- Same **protein family** (e.g., GRK4 and GRK2 are both **G protein-coupled receptor kinases**).
- Structural similarity (e.g., homologous domains, catalytic site conservation).
- Functional similarity (e.g., overlapping substrates).
Output Format

```
json
{
  "Relevant": "True"  // If the target in this BioAssay shares protein family, structure, function, or pathway with the {Query Protein, else set to "False". Only set to "False" if there is no meaningful similarity.
}
```

Query Protein
{protein description}
BioAssay JSON
{BioAssay content}

A.9.4 Ablation study generation prompt

Ablation study prompt

I would like to design drug-like small molecules with high binding affinity to a specific protein. `{protein_description}` Think step by step, generate 10 unique SMILES strings, try to generate diverse molecules instead of making slight change on current molecule. Each molecule should have '[BOS]' at the beginning and '[EOS]' at the end. Each SMILES should be numbered from 1 to 10 and on a separate line.

A.9.5 Molecule optimization prompt

Molecule optimization prompt

To enhance molecular specificity and minimize off-target effects, we aim to reduce potential activity against the hERG channel.
{hERG description}
{hERG BioAssays}
Given the retrieved BioAssays for the hERG channel and the associated activity data table, identify molecular features commonly associated with low activity as favorable and those associated with high activity as undesirable. Using this information, optimize the following ten candidate SMILES strings to reduce their likelihood of interacting with the target. {Input SMILES}
The output should follow the same format: ten optimized SMILES strings, each enclosed in [BOS] and [EOS], with numbering from 1 to 10.
